# The relationship of systemic markers of renal function and vascular function with retinal blood vessel responses

**DOI:** 10.1007/s00417-016-3432-9

**Published:** 2016-07-20

**Authors:** R. Heitmar, C. Varma, P. De, Y. C. Lau, A. D. Blann

**Affiliations:** 1 Aston University, School of Life and Health Sciences, Aston Triangle, B4 7ET Birmingham, UK; 2 Department of Cardiology, City Hospital, Birmingham, B18 7QH UK; 3 Department of Diabetes and Endocrinology, City Hospital, Birmingham, B18 7QH UK; 4 University of Birmingham Institute for Cardiovascular Sciences, City Hospital, Birmingham, B18 7QH UK

**Keywords:** Microcirculation, Retinal vessels, Diabetes, Renal function, Vascular function

## Abstract

**Purpose:**

To test the hypothesis of a significant relationship between systemic markers of renal and vascular function (processes linked to cardiovascular disease and its development) and retinal microvascular function in diabetes and/or cardiovascular disease.

**Methods:**

Ocular microcirculatory function was measured in 116 patients with diabetes and/or cardiovascular disease using static and continuous retinal vessel responses to three cycles of flickering light. Endothelial function was evaluated by von Willebrand factor (vWf), endothelial microparticles and soluble E selectin, renal function by serum creatinine, creatinine clearance and estimated glomerular filtration rate (eGFR). HbA1c was used as a control index.

**Results:**

Central retinal vein equivalence and venous maximum dilation to flicker were linked to HbA1c (both *p* < 0.05). Arterial reaction time was linked to serum creatinine (*p* = 0.036) and eGFR (*p* = 0.039); venous reaction time was linked to creatinine clearance (*p* = 0.018). Creatinine clearance and eGFR were linked to arterial maximum dilatation (*p* < 0.001 and *p* = 0.003, respectively) and the dilatation amplitude (*p* = 0.038 and *p* = 0.048, respectively) responses in the third flicker cycle. Of venous responses to the first flicker cycle, HbA1c was linked to the maximum dilation response (*p* = 0.004) and dilatation amplitude (*p* = 0.017), vWf was linked to the maximum constriction response (*p* = 0.016), and creatinine clearance to the baseline diameter fluctuation (*p* = 0.029). In the second flicker cycle, dilatation amplitude was linked to serum creatinine (*p* = 0.022).

**Conclusions:**

Several retinal blood vessel responses to flickering light are linked to glycaemia and renal function, but only one index is linked to endothelial function. Renal function must be considered when interpreting retinal vessel responses.

## Introduction

The relatively straightforward non-invasive assessment of the retinal circulation by methods such as fundus photography, video recording and tomography has led to the view that it has potential as a screening tool for cardiovascular disease such as myocardial infarction and stroke [1–3]. This concept has been supported through large population studies [4–6] that together demonstrate a relationship between certain retinal vessel indices and cardiovascular risk [7, 8]. Individuals with good cardiovascular health are less likely to have signs of retinopathy such as dilated retinal venules and narrow retinal arterioles, both of which are associated with increased risk of stroke and coronary artery disease [9].

Whilst retinal vessel calibres provide only static indices, dynamic measurements such as retinal vessel reactivity to flicker light provocation can provide further insight into the status of the retinal microcirculation. Several authors have demonstrated a link between measures of cardiovascular health and retinal vessel dynamics, such as a decrease in retinal vessel dilation in the presence of decreased flow mediated dilation of the brachial artery [10], prolonged reaction times in retinal arterial responses to flicker light in patients suffering from coronary artery disease [11], and decreased vessel dilation to flicker light in patients with coronary artery disease, and which depends on the severity of the disease [12]. There is considerable evidence that retinal vessel reactivity to flicker light provocation is blunted in the presence of diabetes and in diabetic retinopathy [13–16].

The classical risk factors for the development of cardiovascular disease operate at the level of the endothelium [17, 18]. Leading plasma markers of endothelial perturbation, known to be abnormal in cardiovascular disease, include von Willebrand factor, soluble E selection and endothelial microparticles [19–21]. A further complicating pathogenic process in cardiovascular disease is poor renal function, known to be present in hypertension and diabetes [22–24], and which is a risk factor for major cardiovascular disease [25, 26]. We therefore hypothesised that retinal arterial and venous static and flicker responses are influenced by renal and/or vascular function. We tested our hypothesis in a clinically relevant cohort of patients with diabetes and/or cardiovascular disease.

## Materials and methods

### Subjects

We recruited 116 patients from out-patient clinics at a University Teaching Hospital. Inclusion criteria were history of cardiovascular disease (myocardial infarction, stroke, >50 % stenosis of artery proven by transcutaneous intervention, artery bypass grafting, amputation) and diabetes (HbA1c > 55 mmol/mol and/or attendance at a diabetes clinic). Exclusion criteria were age <18 years, connective tissue disease, cancer, recent (<3 months) cardiovascular events such as myocardial infarction or stroke, recent (<3 months) surgery, established ocular disease such as age-related macular degeneration. Ethical approval was obtained from West Birmingham Ethics Committee and Aston University Ethics Committee. Written informed consent was received from all individuals taking part in the study. This study has been designed and conducted in accordance with the Declaration of Helsinki.

### Study protocol

A full history and examination took place to ensure that subjects were free from any disease as outlined in the exclusion criteria. All subjects were instructed to refrain from consuming caffeinated products, chocolate, drinking alcohol and smoking on the study day. Intraocular pressure (IOP) was measured by contact tonometry and calculated as the mean of three consecutive readings after instillation of one drop of 0.4 % benoxinate hydrochloride (Chauvin Pharmaceuticals Ltd., Kingston-Upon-Thames, UK; TonopenXL, Medtronic Solan, PMS Instruments, Maidenhead, UK). Data were discarded if the coefficient of variation (CV) exceeded 5 %.

Dynamic and static retinal vessel assessment was determined after full pupil dilation was reached with 1 % tropicamide (Chauvin Pharmaceuticals Ltd., Kingston-Upon-Thames, UK), digital fundus images and reactivity parameters of retinal blood arteries and veins were obtained (retinal vessel analyser [RVA], Imedos Systems (UG) haftungsbeschraenkt Jena, Germany) [27]. For static vessel analysis, black and white fundus images were obtained at a 30° angle with the optic nerve head centred using the inbuilt Zeiss 450 F fundus camera (Zeiss GmbH, Germany). Arterial and venous diameters provided an arteriovenous ratio (AVR), central retinal artery equivalent (CRAE) and central retinal vein equivalent (CRVE; Vesselmap software, Imedos Systems (UG) haftungsbeschraenkt, Jena, Germany) [28, 29]. CRVE, CRAE and AVR were calculated from arteries and veins that were located within a ring whose centre was the optic nerve head and whose inner and outer margins were of one half disc diameter (DD) and one full DD. These measurements (AVR, CRAE and CRVE) are standard ophthalmological indices and are used to describe the physical structural of different retinal arteries and veins such as luminal diameter.

Static imaging was followed by dynamic assessment where retinal diameters were measured continuously at a sampling rate of 25 Hz. Stimulation of retinal blood vessels was done by optoelectronic interruption of the green fundus illumination used by the RVA resulting in a flickering light provocation with a 12.5-Hz frequency [30–32]. After BP stabilisation and image focussing, a vessel segment of the superior temporal retinal artery and vein (500 μm in length) was chosen at a distance of 1.5–2 DD away from the margins of the optic nerve head. Baseline diameter of both the artery and vein was recorded according to the standard RVA protocol [32] for 50 s and then followed by 3 cycles of 20-s flicker provocation with each 80-s recovery time. This resulted in a 350-s measuring period during which the fellow eye was occluded. From the diameter recordings, the values for maximum dilation (MD), maximum constriction (MC) and dilation amplitude (DA), arterial baseline corrected flicker response (BFR), and arterial and venous reaction time (RT) to flicker provocation were calculated [33].

### Plasma markers

Venous blood was collected into citric acid and plasma obtained after centrifugation at 1000 g for 20 min. Von Willebrand factor (vWf) and soluble E selectin were measured by commercial enzyme linked immune-sorbent assay (ELISA; Dako-Cytomation, Ely, Cambs UK and R&D Systems, Abingdon, UK). The ELISAs had intra- and inter-assay coefficients of variation <5 % and <10 %, respectively. Endothelial microparticles were determined by flow cytometry (Apogee Flow Systems, Hemel Hemstead, UK) using fluorochrome-linked monoclonal antibodies to CD144 (R&D Systems, Abingdon, UK) [34]. The size of the EMP was confirmed with polystyrene beads of 110-, 200-, 500-nm and 1-μm diameter together with 300- and 880-nm silica beads (Apogee Flow Systems, Hemel Hemstead, UK). Creatinine and HbA1c were measured by standard techniques by the Hospital Routine Pathology Laboratory. Creatinine clearance and the estimated glomerular filtration rate (eGFR) were calculated according to the Cockcroft and Gault, and the Modification of Diet in Renal Disease equations, respectively [35, 36].

## Statistics

We hypothesised that renal function (as defined by creatinine clearance, serum creatinine and eGFR), systemic endothelial function (as marked by endothelial microparticles, von Willebrand factor and soluble E selectin) and glycaemia (as assessed by HbA1c) each have an effect on retinal vessel function. We tested these hypotheses in a multi-variate linear regression analysis taking each ocular index and the dependent variable and the seven research indices as independent variables. According to Altman [37], a sample size of at least ten is required for each independent variable, of which we have seven, thus calling for a total sample size of at least 70 patients. However, in view of the possible likelihood of a relationship between the seven indices, and in order to obtain greater confidence we decided to over-recruit by at least 50 % (i.e. to at least 105 patients), eventually recruiting 116 patients. Continuously variable data are presented as mean and standard deviation or as median and interquartile range as distribution demands, and were correlated by Spearman’s method. Categorical data are presented as number and percentage. Analyses were performed on Minitab version 17 (Minitab Inc, Coventry, UK).

## Results

Tables 1, 2 and 3 show the clinical, demographic and ocular indices of all 116 patients. Median duration of disease in the 73 diabetics was 10 years (interquartile range 4.5–16.5 years).

**Table 1:** Clinical data, medication and demographics

Feature	Data	Feature	Data
Demographics			
Age [years]	64.3 (9.9)	Sex [m/f]	86/30
Weight [kg]	86.6 (16.6)		
Clinical			
BMI [kg/m^2^]	29.3 (5.5)	SBP [mm Hg]	126 (16)
DBP [mm Hg]	73 (11)	HR [bpm]	72 (13)
Co-morbidities			
Coronary artery disease [n,%]	61, 52.6 %	Peripheral artery disease [n, %]	9, 7.7 %
Cerebrovascular disease [n,%]	11, 9.5 %	Diabetics [n, %]	73, 62.9 %
Medications			
Calcium channel blockers [n,%]	47, 40.5 %	ACEI/ARB [n, %]	84, 72.4 %
Lipid-lowering [n,%]	103, 88.8 %	Aspirin [n, %]	76, 67.8 %
Clopidogrel [n,%]	15, 12.9 %	Nitrate [n, %]	22, 19.0 %
Oral anticoagulant [n,%]	16, 13.8 %	Beta blocker [n, %]	51, 44.0 %
Diuretic [n,%]	45, 38.8 %	Thyroxine [n, %]	15, 12.9 %
Metformin [n, %]	47, 40.5 %	Sulphonylurea [n, %]	14, 12.1 %
DPP-4 inhibitor [n,%]	14, 12.1 %	Insulin [n, %]	29, 25.0 %
GLP-1 agonist [n,%]	8, 6.9 %	Piaglitazone [n, %]	6, 5.2 %

*M* Male; *f* female; *SBP* systolic blood pressure; *DBP* diastolic blood pressure; *HR* heart rate; *BMI* body mass index; *HbA1C* glycated haemoglobin; *DM* diabetes mellitus; *ACEI* angiotensin-converting-enzyme inhibitor; *ARB* angiotensin receptor blockers; *DPP-4* dipeptidyl peptidase-4 (‘gliptins’); *GLP-1* glucagon-like peptide-1 (exenatide, liraglutide). Data presented as mean with standard deviation, or as number of subjects and percentage

**Table 2:** Laboratory indices

Index	Data
Renal markers	
Serum creatinine (μmol/L)	97 (32)
eGFR (ml/min/1.73)	69 (18)
Creatinine clearance (ml/min)	85 (30)
Vascular markers	
Von Willebrand factor (IU/dL)	114 (22)
Endothelial microparticles (×10^3^/μL)	35.9 (9.4–91.1)
Soluble E selectin (ng/mL)	25 (9.5)
Glycaemic marker	
HbA1c (mmol/mol)	53 (17)

*eGFR* Estimated glomerular filtration rate. Data presented as mean and standard deviation or as median and interquartile range

**Table 3:** Ocular indices

(a) Resting ocular indices and Imedos indices				
Index			Data	
Intraocular pressure (mm Hg)			14.1 (2.5)	
Central retinal artery equivalent (arbitrary units)			176 (17)	
Central retinal vein equivalent (arbitrary units)			212 (19)	
Artery/vein ratio			0.83 (0.09)	
Artery diameter (μm)			113 (19)	
Vein diameter (μm)			141 (19)	
Imedos indices:				
Amax - Maximum arterial dilation after flicker (%)			0.9 (0.1–2.0)	
Amin - Minimum arterial dilation after flicker (%)			−0.5 (−1.1–0.1)	
Apeak – Arterial dilation amplitude (%)			1.4 (0.3–2.9)	
Vmax – Maximum venous dilation after flicker (%)			2.8 (1.7–3.9)	
(b) Retinal vessel flicker indices				
	Flicker 1	Flicker 2	Flicker 3	Averaged flicker response
Arterial responses				
Maximum dilatation (%)	3.8 (3.5)	3.6 (3.2)	4.0 (3.4)	2.8 (2.1)
Maximum constriction (%)	−2.2 (1.9)	−2.4 (1.9)	−2.1 (1.8)	−1.9 (1.4)
Dilatation amplitude (%)	5.1 (3.3–7.7)	5.0 (3.1–7.7)	5.1 (3.2–7.5)	4.0 (2.6–6.4)
Reaction time (seconds)	18 (10–22)	18 (13–26)	19 (14–31)	18 (12–24)
Venous responses				
Maximum dilatation (%)	4.4 (2.1)	4.8 (2.5)	4.7 (3.0)	4.3 (2.0)
Maximum constriction (%)	−1.3 (1.8)	−1.1 (2.0)	−1.1 (2.4)	−0.8 (1.3)
Dilatation amplitude (%)	5.0 (3.9–7.3)	5.4 (4.1–7.4)	5.2 (3.6–7.3)	4.6 (3.5–6.2)
Reaction time (seconds)	20 (17–23)	20 (15–23)	20 (15–23)	20.4 (6.7)

Summarized retinal vessel calibres and software-generated vessel reactivity parameters and IMEDOS software-generated arterial and venous responses to flicker. Data presented as mean (SD) or median (lower quartile-upper quartile)

In the analyses of the ocular indices of Table 3(a), although intraocular pressure was linked to creatinine clearance (*p* = 0.014), eGFR (*p* = 0.004) and serum creatinine (*p* = 0.011) in univariate analysis, none remained significant in multivariate analysis. HbA1c was linked to CRVE (*p* = 0.014) and venous size (*p* = 0.034), but there were no other links with other venous indices or any arterial index. In analysis of the IMEDOS indices, no laboratory or clinical index was significantly linked to any arterial index, but V max was linked to HbA1c (*p* = 0.038). Regarding the ocular indices in Table 3(b), no laboratory or clinical index was linked to averaged arterial maximum dilatation, constriction or dilatation amplitude or baseline diameter fluctuation, but in univariate analysis, the arterial reaction time was linked to serum creatinine (*p* = 0.033) and the eGFR (*p* = 0.035), and both were retained in multivariate analysis (*p* = 0.036 and *p* = 0.039, respectively). Of the averaged venous indices, once more, maximum dilatation, constriction or dilatation amplitude or baseline diameter fluctuation failed to link to any index. However, venous reaction time was linked to creatinine clearance (univariate *p* = 0.024, multivariate *p* = 0.018) and eGFR (univariate *p* = 0.033 but multivariate *p* = 0.126).

Correlations between the laboratory indices are present in Table 4. Von Willebrand factor correlated with soluble E selectin, whilst (unsurprisingly) the three renal indices strongly inter-correlated. For each ocular index, we first performed a univariate analysis of all seven research indices, then a multivariate analysis with only those indices that were significant (*p* < 0.05) in the univariate analysis. Analyses of the arterial and vein responses to the three individual flicker cycles in relation to the laboratory and haemodynamic indices are presented in Table 5. The creatinine clearance and eGFR were both independently linked to the arterial maximum dilatation response and the dilatation amplitude response to the third flicker cycle. As regards venous responses, in the first flicker cycle, HbA1c was linked to the venous maximum dilation response and dilatation amplitude, von Willebrand factor was linked to maximum constriction, and creatinine clearance was linked to baseline diameter fluctuation.

**Table 4:** Correlations between laboratory indices

	HbA1c	Creatinine clearance	Serum creatinine	Estimated GFR	Endothelial microparticles	Von Willebrand factor
Soluble E selectin	−0.022	−0.01	−0.085	0.078	0.083	0.401
	0.823	0.924	0.389	0.427	0.394	<0.001
Von Willebrand factor	0.127	−0.046	0.120	−0.047	0.034	
	0.178	0.658	0.200	0.614	0.721	
Endothelial microparticles	0.098	0.092	0.013	−0.005		
	0.302	0.381	0.892	0.956		
Estimated	−0.013	0.733	−0.852			
GFR	0.172	<0.001	<0.001			
Serum	−0.155	−0.55				
Creatinine	0.102	<0.001				
Creatinine	−0.139					
Clearance	0.185					

Data are Spearman correlation coefficient and *p* value. *GFR* Glomerular filtration rate

**Table 5:** Univariate and multivariate links between arterial and venous flicker responses and laboratory and haemodynamic indices

	Flicker 1	Flicker 2	Flicker 3
Arterial responses			
Maximum dilatation (%)	None	None	Cr Cl: <0.001–<0.001 Creat: 0.072–0.117 eGFR: 0.002–0.003
Maximum constriction (%)	None	None	None
Dilatation amplitude (%)	None	None	Cr Cl: 0.013–0.038 eGFR: 0.022–0.048
Baseline diameter fluctuation (%)	None	None	None
Reaction time (seconds)	None	Creat: 0.023–0.666 sEsel: 0.007–0.590	None
Venous responses			
Maximum dilatation (%)	HbA1c: 0.004–0.004	None	None
Maximum constriction (%)	vWf: 0.016–0.028	None	None
Dilatation amplitude (%)	HbA1c: 0.008–0.017 vWf: 0.019–0.072	Creat: 0.022–0.543	None
Baseline diameter fluctuation (%)	Cr Cl: 0.029–0.043	None	None
Reaction time (seconds)	None	None	None

Data are *p* values from univariate and then multivariate regression analysis. *Cr Cl* Creatinine clearance, *Creat* serum creatinine, *eGFR* estimated glomerular filtration rate, *SBP* systolic blood pressure, *HR* heart rate, *sEsel* soluble E selectin

## Discussion

The eye represents a unique opportunity to non-invasively assess microcirculation, and may be useful in predicting those at risk of cardiovascular disease [2–10, 38–40]. A further development in ocular pathology is the recognition of the value of retinal vessel responses to flickering light in diabetes and cardiovascular disease [14–16, 41–43]. A potential pathophysiological process to explain these abnormal retinal vessel responses is endothelial dysfunction [11–13, 17, 18, 44–46], known to be present in diabetes [47–49]. However, the kidney can also be a target organ in diabetes, and may also be linked to endothelial dysfunction [50–52]. Despite this, the role of renal function in retinal vessel responses to flicker light is unexplored.

We tested the hypothesis that retinal vessel responses in patients with diabetes and/or cardiovascular disease would be linked to vascular and/or renal function, but factored in glycaemia as a reference pathological process. As markers of endothelial function, we chose endothelial microparticles, von Willebrand factor and soluble E selectin [19, 20]. The former are shed from the cell membrane as a result of endothelial injury and in vasomotion disorders involved in cardiovascular disease, including diabetes [21, 53, 54]. Von Willebrand factor, a large multimeric glycoprotein stored in endothelial Weibel–Palade bodies and released in a steady state directly from translation and transcription, promotes platelet–platelet and platelet–subendothelial adhesion, and is a co-factor for coagulation factor VIII [55]. However, at times of increased endothelial stress, damage or activation, Wiebel–Palade bodies release large amounts into the plasma, thereby promoting thrombosis [56]. Increased levels in diabetes and diabetic retinopathy are a long-established fact [57, 58]. The adhesion molecule E-selectin (CD69E) is a membrane component upregulated at times of endothelial activation, under which conditions it mediates adhesion between the endothelium and leukocytes [59]. Serum proteases can generate a cleaved product, i.e. soluble E selectin, increased levels of which reflect increased endothelial activation [60, 61]. Potential mechanisms for increased plasma levels of these markers in diabetes are diverse and include the effects of hyperglyaemia, advanced glycation end products, reactive oxygen species and inflammation [49, 62, 63].

We found that HbA1c is linked to several venous retinal vessel indices — the CRVE, venous size, V max, and the maximum dilatation and dilatation amplitude to the first flicker cycle. No arterial index was linked to HbA1c. Only one vascular marker (von Willebrand factor, known to be increased in diabetes [57, 58, 64]) was linked to a retinal vessel response — that of venous maximum constriction. However, renal markers were linked to retinal vessel responses on several occasions. The arterial reaction time was linked to serum creatinine and the eGFR, and in the third flicker cycle, maximum dilation was linked to creatinine clearance and the eGFR, and dilation amplitude to creatinine clearance and the eGFR. However, venous reaction time was linked to creatinine clearance, and venous baseline diameter fluctuation was linked to creatinine clearance in the third flicker cycle.

Notably, no renal or vascular index was linked to intraocular pressure or to any of the IMEDOS indices, but several indices were linked to flicker response indices. The fact that arterial responses were linked to renal indices in the third, but not the first or second flicker cycles, is curious. This result, remaining present after adjustment for multiple analyses and factors, is robust and presumably reflects a pathophysiological feature of the arterial vessel that is apparent only after serial stimulation. This is counter to the link between vascular, renal and glycaemic indices and venous responses that were evident only in the first flicker cycle, when vessels were relatively unstimulated. We also note that soluble E selectin and endothelial microparticles, both known to be increased in diabetes [47, 53, 54, 65], were not linked to any ocular or retinal vessel index.

The most powerful relationships we found were between the positive correlations between two renal indices and arterial maximum dilation in the third flicker cycle. This we interpret as high maximum arterial dilations, reflecting good microvascular responses, are linked to good renal function as are reflected by high eGFR and high creatinine clearance. This is similar to the link between dilation amplitude and renal indices. The reason why these are present not in the first or second, but only in the third cycle may be that the vessels are less able to control their responses after a prolonged bout of stimulation.

The link between venous responses and HbA1c in the first flicker cycle alone is perhaps unsurprising given the weight of literature on the effect of this risk factor on the vasculature [39–44]. However, this is in contrast to the lack of an arterial link which supports the work of Mandecka et al. [14] who also failed to correlate HbA1c with flicker responses. The links with von Willebrand factor and creatinine clearance, although present and adjusted for multiple analyses, are, nonetheless, weak and so may be spurious.

Our data confirms, contrasts and extends that of others. Yip et al. [66] reported that retinopathy was associated with end-stage renal disease, the latter being unrelated to retinal arteriolar calibre, retinal venular calibre and retinal vascular fractal dimension. Eriksen et al. [67] found that the measured GFR, but not a creatinine-based eGFR, was linked to retinopathy, but not to retinal artery or vein diameters. Our data supports this finding as our creatinine-based eGFR did not correlate significantly with retinal artery or vein diameters. Using retinal fundus photography, Lim et al. [68] described an association between narrower retinal arteriolar calibre, smaller retinal vascular fractal dimensions and the presence of AV nicking and opacification with lower eGFR and a higher urinary albumin to creatinine ratio and microalbuminuria, the latter reflecting more severe renal disease. They concluded that quantitative changes of the retinal vascular geometry and qualitative changes in the vessel architecture are associated with markers of renal dysfunction and damage. Together, these three sets of data all support, to one extent or another, the general hypothesis that renal function is important in retinal vessel integrity, as does our own data.

We acknowledge the limitation of multiple analyses, and that many of these are likely to be physiologically (the vessel indices) and/or mathematically (the renal indices) related (correlations in Table 4) and so we may be at risk of false positives. However, we feel this is countered by the large sample size and that we have not over-interpreted our data. Our data may also be limited by possible effects of various systemic medications being taken by the patients, and that we recruited from a well-motivated group that may contribute to a better than expected endothelial function compared to poorly motivated patients on different medications. Nevertheless, our population is fully clinical in that all were being seen in a secondary care setting for diabetes and/or cardiovascular disease, and so represent the full spectrum of diabetic atherosclerosis.

We conclude that HbA1c may be an influence on venous responses to a first flicker stimulation, and that renal function is an influence on arterial responses to a third flicker cycle. Flicker stimulation analyses should consider the effects of these risk factors.
